# Case of Spermatorrhœa, Treated by Cauterization of the Urethra

**Published:** 1851-01

**Authors:** J. Root

**Affiliations:** Fon-du-Lac, Wisconsin


ART. IV.—*Case of Spermatorrhœa, treated by cauterization of the urethra.* By J. Root, M. D., ofFon-du-Lac, Wisconsin.

T. C., a young man, aged about 22 years, came under my care while residing in Lockport, N. Y., 25th May last. He had been treated for some time previous for nervous disorders, dyspepsia, sleeplessness, worms, &c., but nothing seemed to relieve the symptoms under which he labored. The impression existed among his friends, that he was laboring under *derangement of mind.* He complained of a very uneasy sensation in the region of the epigastrium, sleepless nights, and a roaring or splashing noise in the head. He appeared to think he was laboring under a very severe form of disease, from which he would never recover. He would rise from his bed in the night, take a light, and look at himself in a mirror, declaring he looked like a dead man; in the day time he would stretch himself on a board and declare he should not move till he was laid in his coffin. He was naturally a very strong, athletic man, of a nervo-sanguineous temperament, accustomed to labor. Stated that he had been ill for some two or three months; previously in good health. His present symptoms had come gradually upon him. His countenance was pale, and haggard, and his eyes, glassy and of a wild expression; pulse quick and feeble; tongue furred; bowels costive. He had no appetite, and had been several days without taking any food. I did not carry my investigation of the case so far, on my first visit, as to come to any understanding of the case. I did not, as I should have done, make any inquiries as to the state of the sexual organs, but I have the poor consolation that others, previously in attendance, were as culpable as myself.

I saw him occasionally, till the 17th of June, prescribing some simple remedies which were of no avail, the patient remaining about the same as when I first saw him.

From a remark of his, at this time, I was led to make inquiries in reference to his sexual habits, and he confessed that he had been subject to masturbation for several years, not knowing, he said, its evil effects.

On examining the penis and testicles, they were very flaccid and diminutive in size. The lips of the meatus urinarius were red and swelled. A

thick, creamy fluid occupied the urethra. He had no erections or sexual desires. On the 18th of June, assisted by my friend, Dr. C. Hill, I applied the nitrate of silver, made into an ointment, to the prostatic portion of the urethra with M. Lallemand's port caustique. No pain followed the operation; perhaps the ointment was too weak. In about a week from the first application, I saw him again. His nights were still sleepless, though his mind was less unhappy. Some erections and sexual desires. Lips of meatus urinarius less red and tumid. On introducing a gum-elastic catheter, the urethra was found more sensitive than previously, and there was some difficulty in passing the prostatic portion. At this time I cauterized the whole length of the urethra, with the solid stick of nitrate of silver. Considerable pain was experienced, and the end of the catheter was covered with blood. No unpleasant consequences followed the operation. In three months after this, the patient was completely restored to health, his symptoms having subsided, and cheerfulness of mind having returned.

I am disposed to think this treatment, recommended by Lallemand, too much neglected. Many of those obscure cases, generally considered incurable, which are passed from quacks to regulars, and back again from regulars to quacks, might, I believe, be successfully treated, if more attention were bestowed on the abuse of the sexual organs. The treatment is simple, and if due caution be observed, safe. Without nauseating drugs or severe regimen, a *cure* can be effected by the simple application of nitrate of silver to the affected parts.

